# Modeling the metachronous ripening pattern of mature green tomato as affected by cultivar and storage temperature

**DOI:** 10.1038/s41598-022-12219-z

**Published:** 2022-05-17

**Authors:** Drupadi Ciptaningtyas, Nisareefah Benyakart, Hitomi Umehara, Masafumi Johkan, Nobutaka Nakamura, Masayasu Nagata, Takahiro Orikasa, Manasikan Thammawong, Takeo Shiina

**Affiliations:** 1 Postharvest and Food Engineering Lab., Graduate School of Horticulture, Chiba University, Matsudo 648, Matsudo City, Chiba 271-8510 Japan; 2 Department of Agricultural and Biosystem Engineering, Faculty of Agroindustrial Technology, Universitas Padjadjaran, Jl. Raya Bandung-Sumedang Km. 21, Jatinangor, 45363 Indonesia; 3 Food Research Institute, National Agriculture and Food Research Organization, 2-1-12, Kannondai, Tsukuba City, Ibaraki 305-8642 Japan; 4 The United Graduate School of Agricultural Sciences, Gifu University, 1-1 Yanagido, Gifu City, Gifu 501-1193 Japan; 5 Faculty of Agriculture, Iwate University, 3-18-8, Ueda, Morioka, Iwate 020-8550 Japan; 6 Faculty of Applied Biological Sciences, Gifu University, 1-1 Yanagido, Gifu City, Gifu 501-1193 Japan

**Keywords:** Applied mathematics, Chemical engineering, Computational models, Plant physiology, Secondary metabolism

## Abstract

Nutritional benefits and organoleptic characteristics, including visual, textural, taste, and flavor, are the critical characteristics of economically important fruit. Ripening is a crucial phenomenon in the formation of these quality characteristics in fruits. Therefore, controlling the ripening phenomenon is extremely important not only to maximize the benefits of the fruit but also to avoid food losses caused by over-ripening. Tomato is an important model plant, especially for research on fruit ripening. The metachronous model of tomato ripening is presented in this report. This model predicts the postharvest ripening time of tomato fruit in terms of red color development based on the storage period. A modified sigmoid-type function model was used to develop the prediction model. The observations and analyses were conducted at different storage temperatures and in different tomato cultivars. The result exhibits that the integration of the proposed model and time lag was successfully showing the postharvest ripening time history of tomato fruit at the full range ripening process, from onset to fully ripe. This study provides critical information on postharvest quality control research and supply chain development in eliminating food loss and waste, which leads to the realization of sustainable development goals.

## Introduction

Tomato (*Solanum lycopersicum* L.) is one of the most economically important herbaceous fruits. It is a widely grown, irrigated crop that has a high nutritive value and may reduce the risk of cardiovascular diseases and some types of cancer^1,2^. The increase in the consumption of fresh tomatoes over the last century has driven commercial demand to improve the postharvest handling by reducing disease proliferation and maintaining the quality of fresh produce during their shelf life^3^.

In an efficient distribution process, tomatoes in good condition can last for several weeks without considerable damage^4^. However, because of the perishability and product variability, physical damage, spoilage, or decay may still occur when inappropriate handling practices are applied in the supply chain^5^. This is a potential risk to food safety because of the increased possibility of contamination by fungal pathogens in spoiled tomatoes^6^.

In Japan, tomatoes are generally harvested at the breaker stage of development. However, in other countries, they are harvested at different maturity stages, including the mature-green stage, to meet the different consumer requirements^7^. Mature-green tomato is physiologically mature but pre-ripe and is green and slightly white in color. Some researchers have reported that harvesting tomatoes at this stage extends their shelf life and facilitates their distribution without damage^8,9^.

Consumers use color and firmness of tomatoes to assess their maturity stage before buying them. As the consumer's preference regarding the maturity stage varies, it is essential to establish a method that can precisely predict the maturity stage of tomatoes based on the storage period. One of the most critical factors affecting the postharvest ripening of tomatoes is the storage temperature as it affects the ethylene production rate. Furthermore, the production rate of ethylene affects the ripening process in tomatoes, which affects red color development. Moreover, preharvest conditions also affect the development of red color during postharvest treatment in tomato^10^.

The mature-green tomatoes stored under the same storage conditions have different onset of red color development because of individual variation. Furthermore, in our previous study^11^, we successfully developed an estimation model to describe the red color development of pericarps of individual tomatoes shown by CIE (*Commission Internationale de l´Eclairage*/ International Commission on Illumination's) a* (negative values denote green and positive values denote red) during ripening as a function of cumulative ethylene production using a sigmoid-type function model at different storage temperature conditions. In the previous study, we also reported that a single sigmoid-type function model based on cumulative ethylene production could cover different patterns of red color development among individual tomatoes. However, measuring the ethylene production rate is not an easy task. Advanced measurement apparatuses are required to obtain high-accuracy data. Furthermore, cumulative ethylene production cannot predict the onset of red color development because the onset of the rise in ethylene production coincides with the onset of the red color development itself.

In the same study, we also observed that there was always the same increasing trend of CIE a* value, following the sigmoid-type function, in relation to the storage period (day) for mature-green stage harvested tomatoes (Momotaro York) after the breaker stage of development. This knowledge evokes the development of a CIE a* value prediction model of mature-green stage harvested tomatoes during ripening, based on the storage period. Therefore, this study aims to develop a novel model for predicting the red color development of tomatoes harvested at the mature-green stage of development during ripening based on the storage period at different storage temperature conditions without any requirement for measurement.

The prediction model of the change in CIE a* value based on the storage period establishes the typical ripening pattern curve of tomatoes. A typical ripening pattern curve is a curve that shows the metachronous phenomenon of postharvest tomato ripening time history. In this study, ripening of tomato was shown by the CIE a* value (red color development). Therefore, the prediction method of the ripening stages of tomatoes harvested at the mature-green stage using the typical metachronous ripening pattern curve at different storage temperatures was executed in this study. The typical ripening pattern curve will support the provision of different maturity stages of tomatoes to meet different consumer requirements. Concurrently, the chances of food loss and waste occurrence as a result of over-ripening will be minimized. This study will contribute to the accomplishment of sustainable development goals.

## Results and discussion

### Change in pericarp color and physical appearance

According to previous studies, the alterations in tomato color are caused by the accumulation of pigments, such as betalains, carotenoids, anthocyanins, and flavonoids, in the peel^12–14^; among them, lycopene is the most prominent pigment^15–17^. Prior to or coinciding with the red color development of tomatoes, degradation of chlorophyll also occurs, which causes a decrease in the intensity of green color.

**Figure 1:**
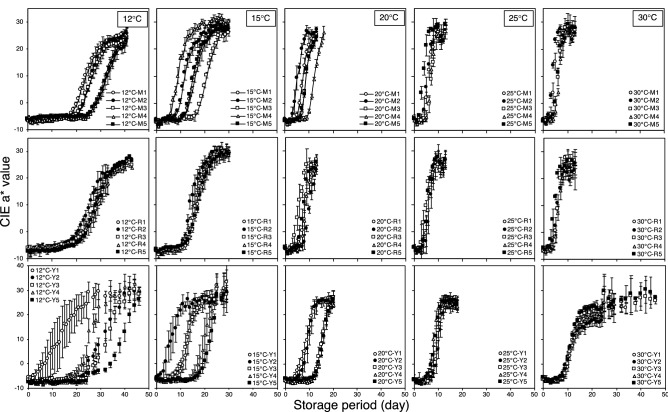
Change in color (CIE a* value) of tomatoes: Miracle (M), Rei-getsu (R), and Momotaro York (Y) harvested at the mature-green stage of development during the ripening at 12, 15, 20, 25, and 30 °C.

Figure 1 shows the change in CIE a* values at ripening for the three tomato cultivars harvested at the mature-green stage at different storage temperatures. The results show that CIE a* values of all cultivars increased from negative to positive values, indicating the red color development. This result demonstrates that Miracle, Rei-getsu, and Momotaro York has undergone a normal postharvest ripening process when it was stored at 12, 15, 20, 25, and 30 °C.

In this study, the equilibrium CIE a* values for most tomatoes were found to be between 24 and 26. A previous study has reported that tomato (*Lycopersicon esculentum*. ‘Caiman’) reaches the fully ripe stage at CIE a* values between 24–26.6^18^. It further indicates that tomatoes in the current study reached the fully ripe stage of development during the storage period at all the tested temperatures. However, in the present study, the equilibrium CIE a* values for cultivars Miracle and Rei-getsu stored at 20, 25, and 30 °C were not established because the CIE a* values at these temperatures did not change after reaching 26.

There is a very close relationship between ethylene release and red color development in tomato fruits^11,19^. This relationship follows the sigmoid function when storage temperatures range from 12 to 25 °C^11^. Furthermore, the ethylene biosynthesis pathway is a temperature-dependent factor^20^, wherein ethylene production levels decrease at low temperatures and vice versa. Temperature triggers the subsequent changes in the ethylene signal transduction pathway in tomatoes. Regarding the close relationship between ethylene release from the tomato fruit and red color development, the phenomenon mentioned earlier explains the various time lag before the onset of red color development, as well as the various times required for tomato to reach the fully ripe stage of development when stored at different storage temperatures. By only considering the storage temperature in this study, the results show that red color development mostly occurs quickly at higher storage temperatures than at lower temperatures.

On average, the onset of the red color development for the three cultivars at 12, 15, 20, 25, and 30 °C occurred around 23, 13, 8, 6, and 6 days of storage, respectively. The fully ripe stage of development at 12, 15, 20, 25, and 30 °C was attained after 39, 21, 14, 10, and 14 days of storage, respectively. Surprisingly, at 30 °C, the onset of red color development and the fully ripe stage occurred simultaneously and were slower than that at 25 °C and 20 °C.

Tomato fruits require optimum storage temperature for lycopene biosynthesis so that the tomato will turn red during the ripening process. Several reports declared that lycopene would only emerge at the storage temperature range between 12 and 32 °C^21–24^. In addition, the tomato will have a mixed color or a speckled color of red, orange, and yellow at 30 °C storage temperature^24^. In this study, the result shows at the same length of the storage period, where the tomatoes stored at 25 °C and 20 °C show a fully red color, the tomatoes held at 30 °C still have orange and yellow colors. The orange and yellow color indicates the existence of β-carotene accumulation since β-carotene will increase at 30 °C even though it will be decreased by the extended storage time^24^. Another report supports this statement that lycopene and β-carotene synthesis will be equally repressed at 33 °C with long-term storage conditions (200 days)^22^.

Genetically, the phytoene synthase mRNA levels paralleled the fruit's color development. The message was present during high storage temperature, however, no change was found in the message level^23^. According to the carotenoid biosynthesis pathway in tomatoes, the high temperature prevents the accumulation of phytoene more than that of lycopene. Since phytoene is the precursor of lycopene, there is no doubt it also affects the accumulation of lycopene in tomato^24^. Moreover, β-carotene is the lycopene successor, produced by the enzyme lycopene β-cyclase^25^. Hence lycopene will be directly used to produce β-carotene without the chance to accumulate as faster as it should be or even has no occasion to accumulate itself at all^25^. Therefore, high storage temperature conditions delayed or even inhibited the red color development of tomato as a ripening parameter^22–24^. In general, normal ripening patterns do not apparently occur at storage temperatures above 30°C^26^. Similarly, even at 30 °C, tomato Cochoro showed slower lycopene accumulation compared to that at 20 °C^27^. This finding holds up the phenomenon where at 30 °C, the onset of red color and fully ripe stage of development were slower than at 25 °C and 20 °C.

Moreover, observations based on the cultivar showed that Miracle is the fastest when it comes to the onset of red color development and ripening speed, followed by Rei-getsu and Momotaro York. Among the three cultivars, the Rei-getsu and Momotaro York showed a slower onset of red color development, as well as ripening speed at 30 °C, than Miracle. This phenomenon caused by a differential response of cultivar for different temperatures for lycopene biosynthesis^27^. According to these findings, the sensitivity of the normal tomato ripening process to high storage temperature varies with the tomato cultivar. Evidently, Rei-getsu and Momotaro York were more sensitive to high temperatures than Miracle in terms of ripening process.

The red color development in all tomatoes stored under different temperatures demonstrates that normal ripening process occurs at these temperatures. However, the results also show that variations exist among individual samples at the same storage temperature.

Even though the onset of red color development was different for the three cultivars, the trend of the change in CIE a* value always followed the sigmoid-type function. This phenomenon reveals the possibility of predicting the ripening stages of tomatoes by estimating the CIE a* value, based on the storage period using a sigmoid-type function model.

### Regression analysis on the sigmoid-type function model

The relationship between change in CIE a* value and storage period of the three tomato cultivars stored at different storage temperatures showed the same S-type trend or the sigmoid-type function curve (Fig. 1). In a previous study, a sigmoid-type function model was used to estimate the ripening stages (CIE a* value) of tomato based on cumulative ethylene production at four different storage temperatures^11^. The estimation model used in the present study is similar to this model except that the free variable is storage period and not the cumulative ethylene production. In the current study, the model is a slightly modified model that was reported for the first time on the sigmoid-type relationship between the CIE a* value and cumulative ethylene production in tomato fruit^19^.

The ripening stages (or the color change) can be predicted by using Eq. (1). However, the modified sigmoid-type function model does not describe the changes in CIE a* value during the green stage (with negative CIE a* value). Therefore, the data were divided into two parts based on the CIE a* value: > 0 (P2) and < 0 (P1).

**Figure 2:**
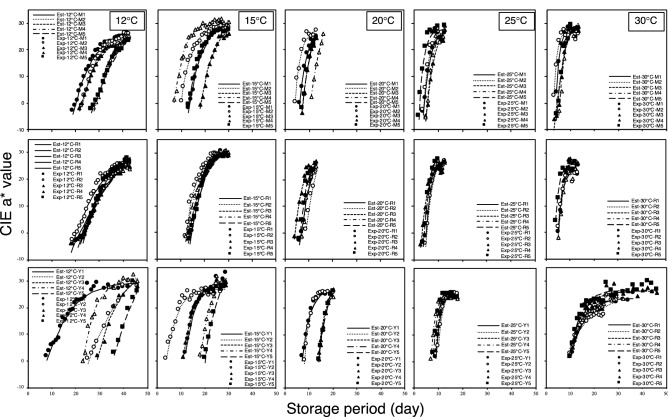
Comparison between experimental and estimated data from Eq. (1) for individual tomatoes of Miracle (M), Rei-getsu (R), and Momotaro York (Y) stored at 12, 15, 20, 25, and 30 °C, based on the proposed sigmoid-type function model.

Figure 2 shows the comparison between the experimental and estimated data from Eq. (1) for individual tomatoes at the different storage temperatures for P2. The results show that the estimated data agrees with the experimental data. The result of the goodness-of-fit shows that the coefficient of determination (R^2^), root mean square error (RMSE), and RMSE (%) ranged between 0.90–1.00, 3.23–0.15, and 0.13%–1.99% respectively (Table 1). This shows that the modified sigmoid-type function model can estimate red color development in mature-green tomatoes during postharvest storage.

**Table 1:** Range value of the coefficient of determination (R^2^), root mean square error (RMSE), %RMSE $\left(\frac{RMSE}{\sqrt{\sum {(CIE \,  a* \, value}^{2})}}\times 100 \right)$, and the average values of parameters α, β, and γ in the equation describing the CIE a* value based on the storage period x [as shown in Eq. (1)]* for tomato cultivars Rei-getsu, Miracle, and Momotaro York stored at 12, 15, 20, 25, and 30 °C.

Cultivar	Storage temperature	R^2^	RMSE	%RMSE	α	β	γ
Rei-getsu	12 °C	0.96–0.99	2.02–1.01	1.04–0.52	292.6	− 0.083	− 256.6
	15 °C	0.98–0.99	1.16–0.70	0.51–0.32	451.8	− 0.189	− 419.5
	20 °C	0.97–0.99	1.64–0.68	1.49–0.62	270.1	− 0.301	− 237.7
	25 °C	0.96–0.97	1.96–1.47	1.34–1.05	294.8	− 0.486	− 267.7
	30 °C	0.96–0.98	1.72–1.01	0.92–0.72	531.8	− 0.627	− 506.8
Miracle	12 °C	0.98–0.99	0.94–0.69	0.51–0.37	639.9	− 0.120	− 608.8
	15 °C	0.98–0.99	1.18–0.54	0.48–0.24	485.6	− 0.217	− 455.4
	20 °C	0.97–0.99	1.26–0.15	1.10–0.13	342.3	− 0.346	− 311.9
	25 °C	0.95–0.96	2.29–1.92	1.42–1.38	237.6	− 0.454	− 208.9
	30 °C	0.94–0.97	3.23–1.25	1.99–0.77	363.9	− 0.580	− 334.6
Momotaro York	12 °C	0.96–0.98	1.73–0.84	0.95–0.46	876.4	− 0.111	− 836.8
	15 °C	0.95–0.98	2.30–0.88	1.12–0.43	776.4	− 0.229	− 744.4
	20 °C	0.97–0.99	1.25–0.78	0.90–0.56	1159.5	− 0.299	− 1127.9
	25 °C	0.97–1.00	1.04–0.90	0.73–0.59	4671.1	− 0.614	− 4645.22
	30 °C	0.90–0.96	1.79–1.08	0.78–0.49	186.7	− 0.193	− 161.8

*$$Estimated \,  CIE \,  {a}^{*} \,  value= \frac{\alpha }{\left(1+{e}^{\beta x}\right)}+\gamma $$.

**Figure 3:**
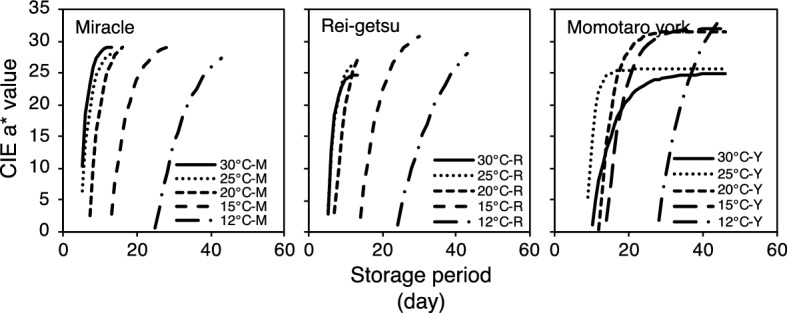
Universal sigmoid-type function curves based on the experimental data that universally describe the red color development of cultivars Miracle (M), Rei-getsu (R), and Momotaro York (Y) stored at 12, 15, 20, 25, and 30 °C.

The average values (Table 1) of parameters α, β, and γ at the same storage temperature were calculated for every tomato cultivar and computed into Eq. (1) to develop a universal sigmoid-type function that can describe the change in the tomato ripening stages at different storage temperatures. Figure 3 shows the universal sigmoid-type function curve based on the experimental data for each cultivar of tomatoes stored at different temperatures. Therefore, the predicted CIE a* value always starts above zero, and the equilibrium CIE a* value for Miracle and Rei-getsu have hardly been outlined.

Determination of the time lag precisely at a CIE a* value equal to zero is crucial so that the proposed model can adequately predict the typical ripening pattern curve of postharvest tomatoes since the onset of red color development. Concurrently, the storage period can be extended in the computation so that the typical ripening pattern curve can describe the complete ripening process of postharvest tomatoes from the onset of red color development until the fully ripe stage of development. These two integrations are discussed in the following two sections.

### Analysis of the time lag

As explained in the previous section, the modified sigmoid-type function model can only describe the change in the positive CIE a* values (P2). Therefore, it is essential to start the estimation model immediately after the CIE a* value turns positive. Theoretically, the breaker stage of development occurs precisely when the CIE a* value is equal to zero. However, experimentally, this value was slightly positive and never exactly zero at the breaker stage of development (Fig. 3). To obtain a precise value for the time lag, the time when the CIE a* value was equal to zero was calculated based on Eq. (2).

**Figure 4:**
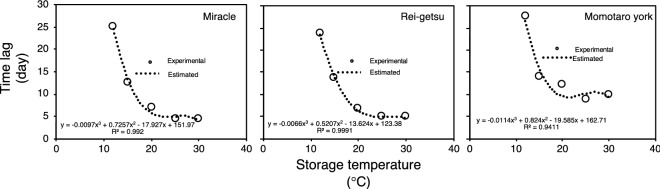
Relationship between time lag of red color development and storage temperature of tomato cultivars Miracle, Rei-getsu, and Momotaro York. Dotted lines denote cubic functions for each variety. Time lag was obtained using Eq. (2) with the average α, β, and γ from five (n = 5) biological replicates.

A clear relationship was observed between the time lag of red color development and storage temperature; it was established using a cubic equation, as shown in Fig. 4. Using this relationship, the time lag of red color development for the three tomato cultivars, stored at any storage temperature between 12 and 30 °C, can be estimated.

### Development of typical metachronous ripening pattern curve

**Figure 5:**
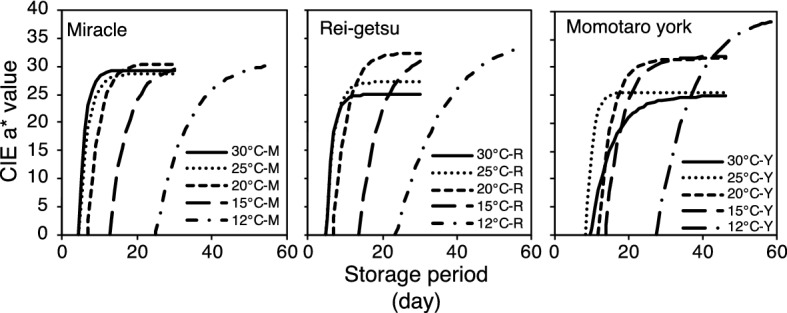
Typical metachronous ripening pattern curve of three tomato cultivars (Miracle, Rei-getsu, and Momotaro York) stored at 12, 15, 20, 25, and 30 °C.

The typical metachronous ripening pattern curve depicts the postharvest color change in mature-green tomatoes at different storage periods by combining the estimated CIE a* value (from the sigmoid-type function model) and the calculated time lag. It shows the change in the CIE a* value at different storage periods. Figure 5 shows the typical metachronous ripening pattern curves of the three tomato cultivars that were stored at different storage temperatures. It describes the complete ripening process of postharvest tomatoes from the onset of red color development to the attainment of fully ripe stage when no noticeable change in the color was detected.

The typical metachronous ripening pattern curve simplifies the previous prediction method, wherein the ripening stages of tomatoes were predicted based on ethylene production^11^. This is the first study to describe the relationship between the CIE a* value and storage period for the full range of ripening process from onset to fully ripe.

By using the typical metachronous ripening pattern curve of mature-green tomato, we can identify different combinations of storage temperature and storage period to achieve a particular stage of ripening. Table 2 shows the required storage period at different temperatures to attain the different ripening stages (breaker, turning, pink, light red, and red) as identified by the United States Department of Agriculture (USDA). Using this information, tomatoes at different ripening stages can be provided to consumers.

**Table 2:** Storage period of three tomato cultivars (Miracle, Rei-getsu, and Momotaro York) stored at 12, 15, 20, 25, and 30 °C to reach the standard ripening stages (breaker, turning, pink, light red, and red) established by United States Department of Agriculture (USDA).

Cultivar	Storage temperature	Storage period (days)				
Miracle	USDA ripening standard					
		Breaker	Turning	Pink	Light red	Red
	12 °C	25	27	31	34	39
	15 °C	13	14	16	18	21
	20 °C	6	7	9	10	12
	25 °C	4	5	7	8	10
	30 °C	4	5	6	6	7
Rei-getsu	USDA ripening standard					
		Breaker	Turning	Pink	Light red	Red
	12 °C	24	26	31	35	39
	15 °C	13	15	18	19	22
	20 °C	6	7	9	11	12
	25 °C	4	5	7	8	11
	30 °C	4	5	6	7	9
Momotaro York	USDA ripening standard					
		Breaker	Turning	Pink	Light red	Red
	12 °C	28	29	32	34	37
	15 °C	14	15	17	18	21
	20 °C	12	13	15	16	18
	25 °C	8	9	10	11	15
	30 °C	10	11	14	17	24

Tomato pictures shown in the table were stored at 15 °C from the respective cultivar.

In this study, a new sigmoid-type function-based prediction model for estimating the ripening pattern of tomatoes was developed for three different cultivars. The proposed modified sigmoid-type function model find the same limitation as to the finding of the previous study^26^, where the long-term ripening prediction is hardly possible because it is difficult to predict the change in color in early green stage before the red color is developed. However, with the application of time lag, it is possible to predict the onset of the red color development itself. Therefore, by combining these two analyses, the full range of postharvest mature-green ripening process of tomato can be predicted. A typical ripening pattern curve was developed from the combination of the proposed model and the time lag.

The typical metachronous ripening pattern curve will enable us to predict the ripening stage of tomatoes, which are harvested at the mature-green stage based on the storage period and temperatures. Additionally, it will promote good practices in the tomato industry because the red color development of harvested tomatoes can be predicted as a function of their storage period. In this study, the prediction model was successfully developed in the static temperature. However, in the real distribution process, tomatoes often experience a change in storage temperature during the supply chain. Therefore, in the future, it is important to develop a ripening prediction model for tomatoes stored at dynamic storage temperature conditions.

## Materials and method

### Plant material and treatment conditions

A total of 75 tomatoes (*Solanum lycopersicum* L.), 25 each from three different cultivars (Miracle, Rei-getsu, and Momotaro York) were selected for this study. Table 3 shows the name of the cultivar, harvesting date, harvesting season, and the greenhouse location where tomatoes were grown. Fruits that were uniform in size (Miracle: 122.4 g ± 11.6 g, Rei-getsu: 146.4 g ± 21.8 g, and Momotaro York: 172.7 g ± 25.0 g) and free from external defects were selected as samples and hand-picked at the mature-green stage of development. The selection of mature green stage tomato as the samples was conducted by using naked eyes. Physically, mature green stage tomato has a green with slightly white color in appearance in the blossom end. Although the standardization was managed based on the naked eyes, the CIE a* value of the selected samples right after harvested were always showed almost uniform CIE a* value, ranging from − 5 to − 6. The samples were numbered and individually packed in an unsealed plastic pouch (20-μm anti-fogging-oriented polypropylene, Heiko. Inc, Yokohama, Japan) to prevent water loss. Then, the fruits were stored at 12, 15, 20, 25, and 30 °C under dark conditions in the incubators (MIR 153, Sanyo Co. Ltd, Tokyo, Japan), with five tomato samples from the same cultivar in each temperature condition.

**Table 3:** Information on cultivars, harvesting date, harvesting season, and greenhouses location of tomato samples.

Cultivar	Harvesting date	Harvesting season	Greenhouse location
Miracle	03-Aug-19	Summer	Kashiwa city
Rei-getsu	03-Aug-19	Summer	Kashiwa city
Momotaro York	20-Nov-19	Autumn	Matsudo city

### Measuring pericarp color and capturing physical appearance

Pericarp color is the most crucial external characteristic for visually assessing the ripeness of tomato fruit^28^. Hence, in this study, a reflectance spectrophotometer (CM-600d, Konica Minolta, Tokyo, Japan), which was calibrated against a white reference plate, was used to determine the pericarp color using the CIE L*, a*, and b* values. The CIE a* value, which denotes colors from green to red, was used to determine the development of red color during storage. The average CIE a* values were calculated at seven locations, three at the fruit equator and fruit shoulder each and one at the fruit blossom end.

Some reports show the fascinating result of the tomato^26^ and cucumber^29^ color prediction model by using CIE a*/b* value as the pericarp color parameter. However, the CIE a*/b* value seems not felicitous for the pericarp color parameter in our developed model. We have compared the goodness of fit of the models that used CIE a* value and CIE a*/b* value as the ordinate, respectively. The result shows that CIE a* value as the ordinate gives better goodness of fit than the CIE a*/b* value. Therefore, we express the pericarp color of tomato in the CIE a* value.

The top and bottom of each tomato were photographed in a photo booth equipped with a digital single-lens reflex camera (D5600, Nikon, Tokyo, Japan) to observe their physical appearance during storage. Changes in pericarp color and appearance were recorded every 24 h until the tomatoes were fully ripe. The analysis was conducted at the laboratory in the Matsudo campus, Chiba University (Matsudo City, Chiba Prefecture, Japan).

### Modeling approach

#### Sigmoid-type function

To estimate the CIE a* value of tomatoes stored at different experimental temperatures, the modified sigmoid-type function model was used, as shown in Eq. (1). In the previous studies, the free variable $x$ denoted the cumulative ethylene production^19^, whereas it represents the storage period in the present study.

1 $$Estimated \,  CIE \,  {a}^{*} \, value= \frac{\alpha }{\left(1+{e}^{\beta x}\right)}+\gamma ,$$

where α, β, and γ are the parameters that should be optimized using a nonlinear least-square method using Solver tools in Microsoft Excel (Version 16.16.27 (201012), https://officecdnmac.microsoft.com/pr/legal/mac/OfficeforMacAttributions.html, Microsoft Corporation, Redmond, WA, USA).

#### Time lag

Time lag is defined as the time (in days) required for the harvested tomatoes to initiate red color development. Experimentally, it was determined based on the days after harvest when tomatoes started developing red color known as breaker stage of development. As a negative CIE a* value denotes green color and a positive value denotes red color, the breaker stage of development will occur precisely when the CIE a* value is equal to zero.

The time lag was estimated using the transformation of Eq. (1). For each cultivar, the day of storage ($x$) when the CIE a* value is equal to zero was calculated by using the average values of parameters α, β, and γ at each storage temperature, as shown in Eq. (2):

2 $$x=\frac{ln\left[\frac{\alpha +\gamma }{-\gamma }\right]}{\beta }$$

#### Fitting approach and goodness-of-fit

A fitting approach was performed using the Solver function in Microsoft Excel to solve the nonlinear curve-fitting problems in the least-square sense and obtain the parameters α, β, and γ. The goodness-of-fit of the proposed mathematical models was evaluated by determining the coefficient of determination R^2^, RMSE, and % RMSE between the predicted and experimental data.
